# Vapour Phase Hydrogenation of Phenol over Rhodium on SBA-15 and SBA-16

**DOI:** 10.3390/molecules191220594

**Published:** 2014-12-10

**Authors:** Liliana Giraldo, Marlon Bastidas-Barranco, Juan Carlos Moreno-Piraján

**Affiliations:** 1 Departamento de Química, Universidad Nacional de Colombia, Bogotá 110911, Colombia; 2 Departamento de Ingeniería Mecánica, Facultad de Ingeniería, Universidad de la Guajira, Riohacha 440001, Colombia; 3 Grupo de Investigación en Sólidos Porosos y Calorimetría, Departamento de Química, Universidad de los Andes, Bogotá 110911, Colombia

**Keywords:** phenol hydrogenation, mesoporous materials, rhodium/SBA, zeolites

## Abstract

In the present work, mesoporous SBA-15 and SBA-16 were synthesised using classical methods, and their physicochemical properties were investigated by X-ray diffraction (XRD), FTIR, TEM and N_2_ adsorption–desorption. Rhodium (Rh, 1 wt %) was loaded on the mesoporous SBA-15 and SBA-16 by an impregnation method. The Rh surface coverage, dispersion and crystallite size were determined by room temperature H_2_ chemisorption on reduced samples. The catalytic activity of Rh supported on mesoporous SBA-15 and SBA-16 was evaluated for the first time in the hydrogenation of phenol in vapour phase in a temperature range between 130 and 270 °C at atmospheric pressure. The reaction over Rh/SBA-15 at 180 °C produced cyclohexanone as the major product (about 60%) along with lower amounts of cyclohexanol (about 35%) and cyclohexane (about 15%). The influences of temperature, H_2_/phenol ratio, contact time and the nature of the solvent on the catalytic performance were systematically investigated. The Rh/SBA-16 system offered lower phenol conversion compared to Rh/SBA-15, but both have a very high selectivity for cyclohexanone (above 60%).

## 1. Introduction

The adequate cleaning of industrial wastewaters is a problem of major concern nowadays. New and more stringent regulations are being imposed, which suggest the need to develop and implement efficacious treatment technologies capable of dealing with the hazardous pollutants present in many industrial waste streams. Phenols and its derivatives are compounds that form part of the reactions for many processes in the pharmaceutical, petrochemical and polymer production industries whose wastewaters may contain phenolic compound concentrations ranging from 500 to 4000 mg·L^−1^ [1,2]. Several techniques are used for the removal of phenolic compounds from water as incineration or biological methods, which have some limitations [3]. Advanced oxidation processes such as wet air oxidation, Fenton, or photochemical processes have been studied extensively, showing some drawbacks or limitations such as the relatively high temperatures and/or pressures needed, large amounts of reagents and complex equipment, *etc*. [4,5,6]. Catalytic hydrogenation of phenol is an emerging and promising alternative attending to environmental criteria since a biodegradable and much less toxic product (*i.e.*, cyclohexanol) can be obtained [7,8]. The hydrotreatment of phenol can follow two routes: hydrogenolysis or hydrogenation, depending on the reaction conditions. More severe conditions (temperatures and pressures higher than 200 °C and 10 bar, respectively) favour hydrogenolysis in which C–OH bonds are broken, yielding benzene, cyclohexene and cyclohexane [9]. When hydrogenation is performed under moderate conditions, cyclohexenol is obtained in a first step. This intermediate readily disappears by two routes, reacting with hydrogen to produce cyclohexanol or evolving via isomerisation to cyclohexanone that can further react with hydrogen to also produce cyclohexanol [10,11]. The product distributions resulting from phenol hydrogenation are a function of the catalyst and the reaction conditions used [12,13,14].

One product of the phenol hydrogenation is cyclohexanone, which is used in the production of caprolactam for the synthesis of nylon-6 [15]. The former route requires high temperatures and pressures and generates undesirable by-products that lower the product yield and complicate the recovery/separation steps. The preparation of cyclohexanone by one-step hydrogenation of phenol is catalysed by Pd supported on Al_2_O_3_, and over MgO-based catalysts [16,17]. The Al_2_O_3_-supported Pd catalysts, however, have a low resistance to deactivation by coke deposition. The use of MgO as a catalyst support on an industrial scale has drawbacks as well, mainly a low surface area and poor mechanical strength. Recently, MgO-Al_2_O_3 _mixed oxides derived from hydrotalcite (HT)-like compounds (MgAl-CHT) have also been used as a support [18,19]. When Pd is supported on a porous material with a certain surface, phenol is adsorbed and receives H_2_ that binds to the aromatic ring following a spill-over mechanism [20,21]. The manner in which the adsorption of phenol occurs varies with the surface chemistry of the solid and this mode directs the product selectivity to cyclohexanone or cyclohexanol. For instance, phenol is adsorbed on acidic supports in a co-planar fashion with respect to the surface, while on basic supports it is adsorbed in a non-planar fashion. Hydrogenation of the former mode of adsorption leads to cyclohexanol preferentially.

Mesoporous materials are inorganic or mixed organic-inorganic solids, which are synthesised by incorporating specific molecular species from a solution into their structure. Their layered or three-dimensional structural frameworks are porous in one or more directions, with correspondingly low densities. Their structural frameworks may be crystalline or amorphous, and the template or structure-directing agent (SDA) is co-crystallised in pores, channels, or interlayers. Additional solvent molecules (usually, but not necessarily water) are also often present in the structure.

Some results have been published on hydrogenation of phenol with different materials. It has been reported that Pd/Al_2_O_3_ and Pd/SiO_2_ catalysts containing Pd nanoparticles in the size range of 3–13 nm were prepared and investigated in a direct selective hydrogenation of phenol to cyclohexanone. Conversion of 99% was then obtained; a selectivity higher than 99% was achieved within 3 h at 333 K [22]. The hydrogenation of phenol in aqueous phase for the study of a continuous trickle bed reactor was used with commercial and some home-made Pd/activated carbon (AC) catalysts for the treatment of phenolic wastewaters [23]. The catalyst of Rh nanoparticles supported on a carbon nanofibre, 5 wt % Rh/CNF, with an average size of 2–3 nm was prepared by a method of incipient wetness impregnation. This catalyst presents a high activity in the ring hydrogenation of phenol in a medium of supercritical CO_2_ (scCO_2_) at a low temperature of 323 K [24]. Other interesting papers on this subject are those that use silica supported Pd and PdAYb catalysts [25] in vapour phase, Pd/Al_2_O_3_-CWE in the hydrogenation of phenol in aqueous phase [26], and selective hydrogenation of phenol to cyclohexanone over palladium supported on calcined Mg/Al hydrotalcite [27].

Some studies on hydrogenation of phenol have been published using rhodium nanoparticles supported on SBA-15 in the presence of supercritical CO_2_ (scCO_2_) where interesting results were obtained because of the ability of scCO_2_ to penetrate inside the meso-channels [28].

The SBA-15 has been used in various catalytic processes such as transesterification of canola oil over sulfated Ti/SBA-15 catalysts for making biodiesel [29], benzene oxidation with ozone [30], hydrolytic hydrogenation of cellulose [31], oxidative degradation of pentabromophenol in the presence of humic substances, and in hydroxylation of phenol by H_2_O_2_ [32].

In fact, a higher hydrogenation rate of propyne to propene has been observed over Pd/ZrO_2_ catalyst compared to Pd/Al_2_O_3_ or Pd/SiO_2_ catalysts [18]. Recently, Ru-supported ZrO_2_ has been found to possess good catalyst activity in the selective hydrogenation of benzene to cyclohexene [19]. In order to obtain materials with a high surface area and ordered pore structures, many research efforts are currently underway on the synthesis of mesoporous type SBA-15 and SBA-16 zeolites using various synthesis pathways [16,17,18]. Although several approaches have been suggested to synthesise SBA zeolites, the retention of a regular pore structure after calcination has been observed. The structural properties of the SBA support also play an important role in the synthesis of catalysts. To obtain a stable mesoporous SBA-15 and SBA-16support, a good interaction between the inorganic species and metal should be assured. It is generally accepted that the use of a high surface area mesoporous support, rather than a commercial, low surface area support, for precious metals or transition metals has some beneficial effect on the catalytic performance. The mesoporous solids have a higher dispersion of reduced metal particles and, as a consequence, would show an improved catalytic performance [26,27,28,29,30,31,32]. 

The aim of the present study is to investigate the use of SBA-15 and SBA-16 as supports for Rh for the catalytic hydrogenation of phenol. The influences of temperature, H_2_/phenol ratio, contact time and the nature of the solvent on the catalytic performance were analysed. Finally, experimental results are compared with the corresponding ones obtained using Pd supported on Al_2_O_3_, MgO, and MgAl-CHT—supports possessing only acid–base properties.

## 2. Results and Discussion

### 2.1. Characterisation of SBA-15 Support and Rh/SBA-15 Catalysts

#### 2.1.1. Nitrogen Physisorption

**Figure 1:**
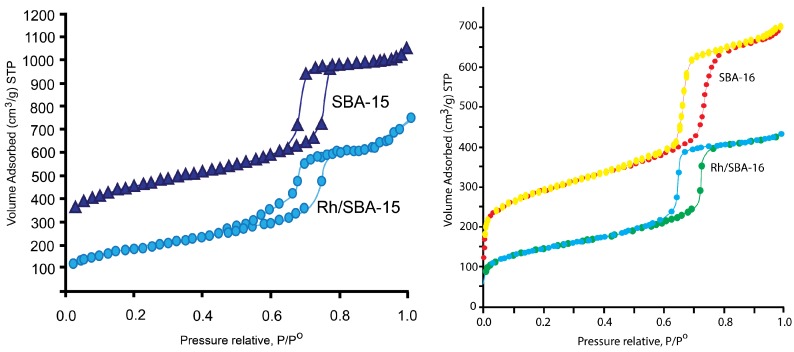
Nitrogen adsorption/desorption of SBA-15 and Rh/SBA-15 catalyst (1 wt %) and N_2_ sorption isotherms of the samples SBA-16 and Rh/SBA-16 (1 wt % in Rh).

**Figure 2:**
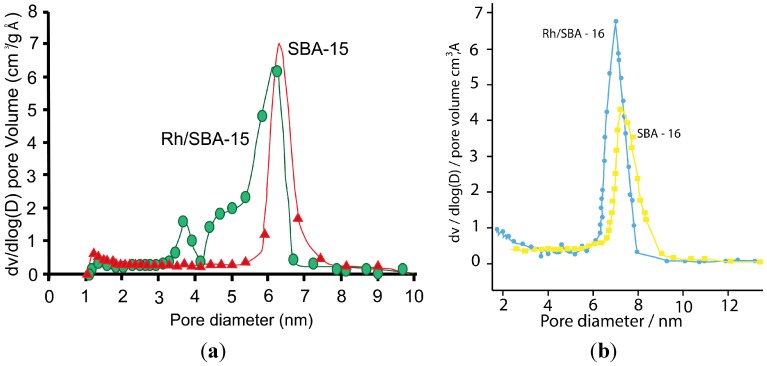
Pore size distribution of (**a**) SBA-15 compared with Rh/SBA-15 (1 wt %) and (**b**) SBA-16 compared with Rh/SBA-16.

Figure 1 shows the nitrogen adsorption–desorption isotherms for the SBA-15 support and Rh-impregnated SBA-15 catalyst (Rh/SBA-15), while Figure 2 depicts the corresponding pore size distributions for these materials using the BJH theory [33,34,35,36,37,38]. 

The obtained isotherms are typical for well-defined mesoporous frameworks [35,36,38]. The shape of SBA-15 and Rh/SBA-15 (1 wt % in Rh) isotherms (Figure 1) corresponds to type IV according to IUPAC classification, and displayed a broad H1 type hysteresis loop characteristic of large pore mesoporous solids [37,38,39]. The initial increase in adsorption capacity at low relative pressure is due to monolayer adsorption in the mesopores. The upward deviation in the range of P/P^0^ = 0.50–0.75 is associated with the progressive filling of the mesopores. As the relative pressure increases, all the isotherms display a slope increase characteristic of capillary condensation inside uniform mesopores. The SBA-15 silica material exhibited a narrow pore size distribution comparable to the results reported in the literature for this type of material [40,41,42]. The capillary condensation step on the nitrogen adsorption isotherm of Rh/SBA-15 (1 wt %) was found to be broad, indicating a wider range of pore sizes, as confirmed by the BJH pore size distribution (Figure 2). Figure 2 displays N_2_ pore size distributions of SBA-15 and Rh/SBA-15 (1 wt % in Rh) samples. Indeed, the pore size distribution of Rh/SBA-15 (1 wt %) shows a peak at 6.8 nm. Furthermore, the Rh-containing SBA-15 retained the SBA-15 mesoporous structure, showing a relatively high specific surface area indicating that this catalyst retained the well-ordered 2D hexagonal array of the SBA-15 support. This suggests that the deposition and impregnation of Rh particles within the mesoporous silica did not seriously affect or block the mesoporous framework channels. Table 1 summarises the textural properties of the SBA-15 support as well as the Rh/SBA-15 catalyst. It is shown that the BET surface area of SBA-15 is 910 m^2^·g^−^^1^ with a total pore volume of 1.39 cm^3^·g^−^^1^ and 7.2 nm pore size Moreover, a moderate decrease in the BET surface area and pore volume of Rh/SBA-15 catalyst is observed.

Similarly, the SBA-16 and Rh/SBA-16 samples exhibited a type-IV isotherm pattern with an H2 hysteresis loop between the partial pressure P/P^0^ = 0.60–0.8 (Figure 2a) for Rh/SBA-16 and P/P^0^ = 0.65–0.85. It is shown that Rh/SBA-16 maintained the mesoporous structure of the parent SBA-16. The BET surface area changed from 655 to 575 m^2^·g^−1^, the pore volume from 1.01 to 0.68 cm^3^·g^−1^ and pore size from 7.3 to 7.6 nm. The significant decreases in these physical parameters imply that rhodium atoms are introduced into the structure of SBA-16.

**Table 1:** Textural properties of catalysts with SBA-15 and SBA-16.

Catalyst	S_BET_ (m^2^ g^−1^)	D_BJH_ (nm)	V_BJH_ (cm^3^ g^−1^)
Rh/SBA-16	575	7.3	0.68
SBA-16	655	7.6	1.01
Rh/SBA-15	625	6.8	1.03
SBA-15	910	7.2	1.39

S_BET_, specific surface area (m^2^·g^−1^); V_BJH_, pore volume (cm^3^·g^−1^); D_BJH_, pore diameter (nm).

#### 2.1.2. Small and Wide Angle XRD 

The XRD patterns (not shown here) indicate that the rhodium-immobilised SBA-15 has an ordered structure. They also show three well-resolved peaks characteristics of this type of catalyst in which the metal structure is maintained once it is deposited in the solid. Rhodium is probably what forms a complex with the free electrons of the structure of SBA-15, obtaining a organised solid. The XRD pattern showed of the calcined sample exhibits three diffraction peaks corresponding to d (1 0 0) of 15.3 nm, d (1 1 0) of 8.3 nm and d (2 0 0) of 5.4 nm, which are a typical SBA-15 XRD reflection pattern. The incorporation of RhCl_3_ causes a marked intensity decrease of the three peaks. However, the basal (100) diffraction peaks are clearly observed. This indicates that the structure of SBA-15 remains intact through the supporting procedure. This is in good agreement with literature [40,41,42,43].

Similarly, the XRD analysis patterns of Rh/SBA-16 (not shown here) displayed a strong diffraction peak of the parent material SBA-16 at a low angle, suggesting that the highly ordered cubic mesoporous structure of SBA-16 was retained during the catalyst preparation. The low angle XRD patterns of SBA-16 were displayed in Figure 2. The SBA-16 exhibits two diffraction (110) and (200) peaks, characteristic of mesoporous materials [44].

#### 2.1.3. Transmission Electronic Microscope (TEM)

**Figure 3:**
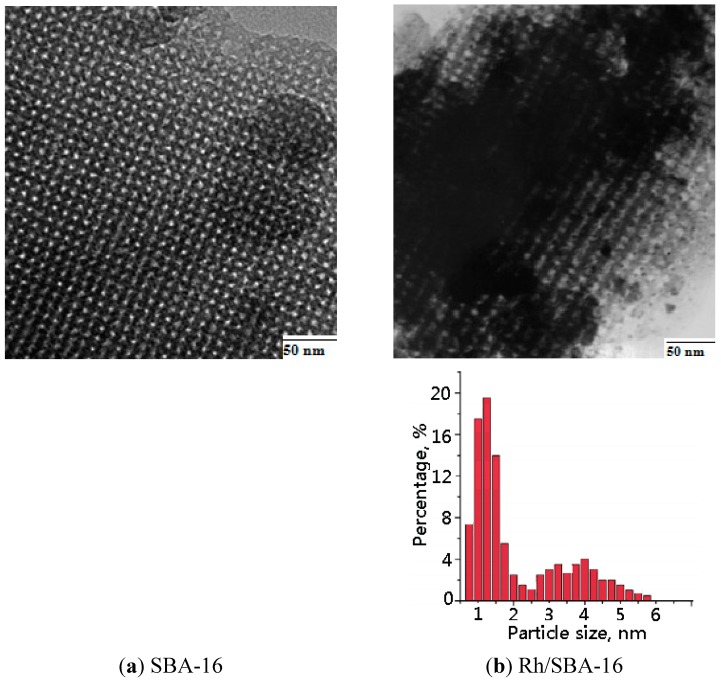
TEM micrographs of (**a**) SBA-16 silica material; (**b**) Rh/SBA-16 catalyst.

In order to analyse the penetration into the SBA-16, we employed TEM to check the Rh particle sizes on the Rh/SBA-16 catalyst with a Rh loading of 1 wt %. As shown in Figure 3a,b, the TEM images clearly display that the ordered pore structure of SBA-16 was retained after the catalyst preparation, and fine Rh particles were located inside the structure of SBA-16 with a narrow pore size distribution. Figure 3b shows the particle distribution of the Rh on SBA-16; particle size was in a range of 1–5 nm and most of them have a size between 1–2 nm. 

**Figure 4:**
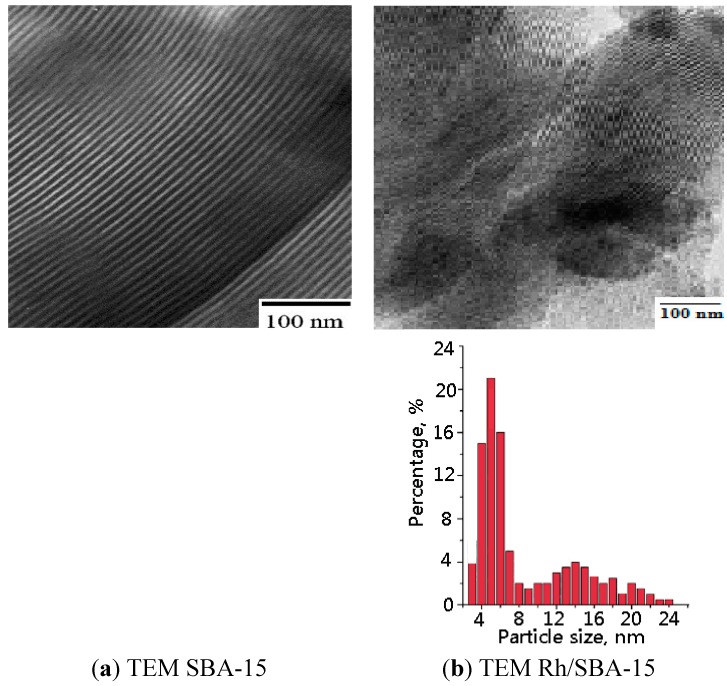
TEM micrographs of (**a**) SBA-15 silica material; (**b**) Rh/SBA-15 catalyst.

The TEM images of SBA-15 and Rh/SBA15 catalysts are displayed in Figure 4. In the Rh/SBA-15 sample (Figure 4b), some small Rh-species attached to the siliceous walls seem to be present. The dispersion of Rh is related to the precursor molecules’ characteristics. Indeed, these precursors can be easily diffuse into the SBA-15 tunnels around 7 nm in size and can be dispersed over the walls of the supporting materials. Figure 4b shows the particle distribution of the Rh on SBA-15; particle size was in a range of 4–8 nm and most of them have a size between 4–5 nm.

#### 2.1.4. Fourier Transform Infrared Spectroscopy (FTIR)

**Figure 5:**
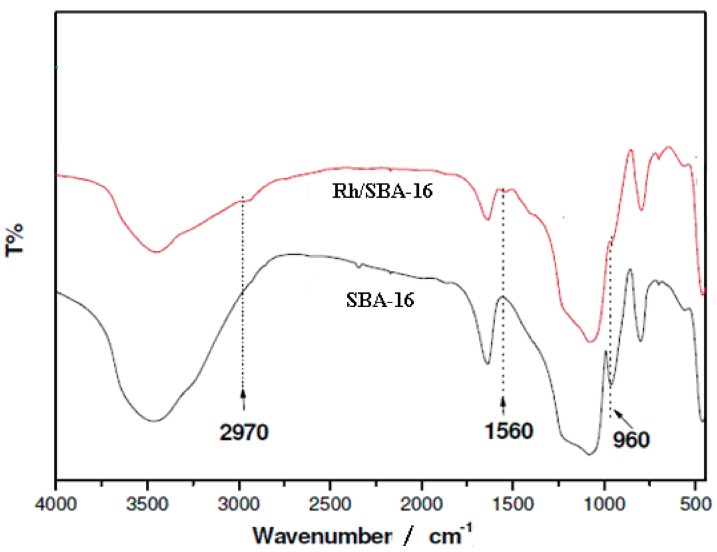
FT-IR spectra od SBA-16 and Rh/SBA-16.

The FT-IR spectra of SBA-16 and Rh/SBA-16 samples are shown in Figure 5. In comparison with the parent material SBA-16, two new peaks appeared at 2970 and 1560 cm^−1^ after modification with amino groups, which correspond to the stretching vibrations of the C–H and the bending vibrations of N–H, respectively (the band at 1640 cm^−1^ was related to the water absorbed on solid surface). These results can confirm that amino groups were successfully grafted on SBA-16. Additionally, after modification, the intensity of the peak at 960 cm^−1^ corresponding to the bending vibrations of Si–OH decreased significantly, proving that the amino groups were covalently linked with SBA-16 via a silylation. Simultaneously, a slight change in the remaining bands due to the presence of rhodium is observed [45,46].

In the summary, both samples show typical bands at 1100 and 900 cm^−^^1^ in the skeletal region of framework vibrations, which are assigned to the asymmetric and symmetric stretching vibrations of Si–O–Si bridges, respectively [45]. In the hydroxyl region, the weak band at 1670 cm^−^^1^ and the broad band at 3500 cm^−^^1^ can be attributed to a combination of the stretching vibration of silanol groups with crosshydrogen bonding interactions and the H–O–H stretching mode of physisorbed water [45]. 

**Figure 6:**
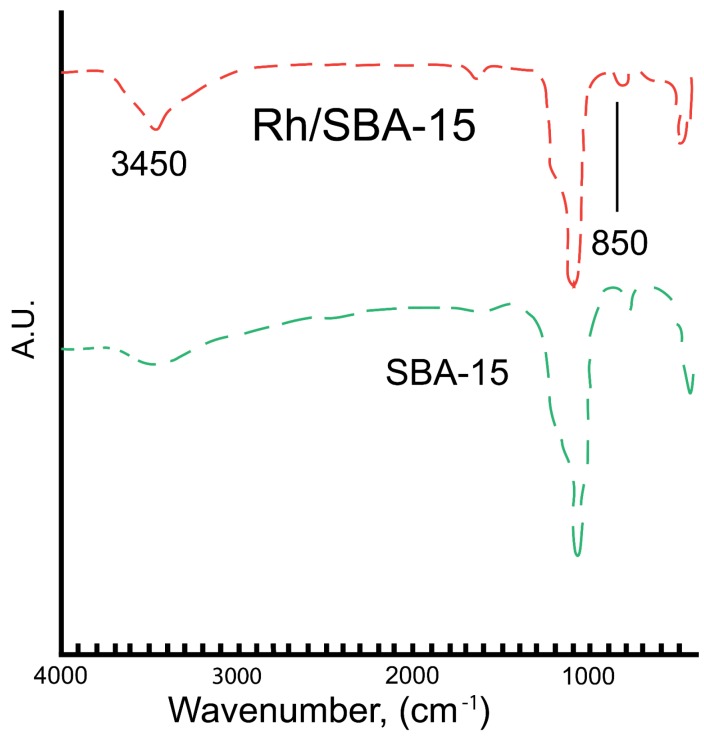
FT-IR spectra of SBA-15 and Rh/SBA-15.

The Fourier transform infrared (FT-IR) spectra of pure SBA-15 and Rh-SBA-15 composite materials are shown in Figure 6. The bands around 3445 cm^–1^ can be assigned to the O-H stretching vibrations mode of the silanols involved in hydrogen interactions with the adsorbed water molecules. The bands at around 1080 cm^–1^ and 850 cm^–1^ corresponded to characteristic anti-symmetric vibrations of non-bridging oxygen atoms (Si–O^δ−^) of Si-O-H bonds and symmetric stretching vibrations (Si–O–Si)_sym_ of tetrahedral SiO_4_ structure units. Meanwhile, the band at around 460 cm^–1^ corresponds to the characteristic tetrahedral bending of Si-O bonds. No typical IR band located at around 960 cm^–1^ can be observed for the pure SBA-15. However, Rh-SBA-15 exhibits practically the same bands, shifting slightly in intensity [45,46,47]. Incorporation of the metal ion in the silica is observed around 960 cm^-1^ which corresponds to the vibration of Si-O affected by the presence of the ion [45,46].

### 2.2. Hydrogenation of Phenol over Rh/SBA

**Figure 7:**
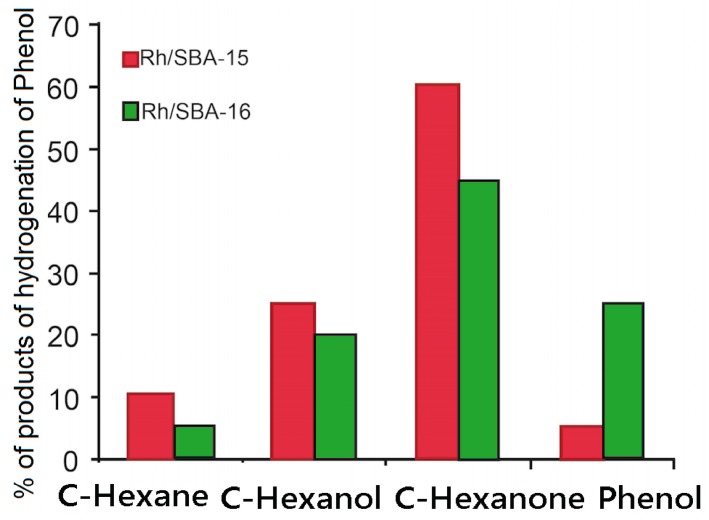
Conversion of phenol with Rh/SBA-15 and Rh/SBA-16.

The hydrogenation of phenol over Rh/SBA-15 and Rh/SBA-16 produced a mixture of cyclohexanone, cyclohexanol and cyclohexane. The phenol conversion and the product selectivity depend strongly on the type of catalyst used and on the reaction conditions used. Figure 7 illustrates the effect of the catalyst on phenol conversion. The phenol conversion reaches 95% for Rh/SBA-15 and 75% for Rh/SBA-16. This result can be closely related to the amount of chemisorbed H_2_ (0.038 mmol·g^−1^ for Rh/SBA-15 and 0.026 mmol·g^−1^) and, then, to the Rh metal surface coverage. 

Reaction conditions: =180 °C. benzene/phenol = 2.0; H_2_/phenol = 6; weight catalyst = 0.6 g; WHSV = 27 mol·h^−1^·kg^−1^ (catalyst)^−1^.

Thus, the catalytic activity for phenol conversion increases with the type of SBA used as support for Rh and also with the quantity of Rh metal atoms exposed on the surface of the SBA. In summary, Figure 7 shows that the % conversion for Rh/SBA-15 and Rh/SBA-16 catalysts have the following behaviour, respectively: 

C-Hexanone > C-Hexanol > C-Hexane > Phenol

C-Hexanone > Phenol > C-Hexanol > C-Hexane

The maximum conversion percentage corresponds to C-Hexanone in both catalysts, being higher for Rh/SBA-15. It is observed that the major product of the hydrogenation of phenol is cyclohexanone; a marked difference between the amount of cyclohexanone produced by the catalyst Rh/SBA-15 (60%) and the Rh/SBA-16 (45%) is observed. A similar behaviour is observed for the other subproducts detected in this investigation. Undoubtedly, both catalysts produce a hydrogenation of phenol that is highly selective for cyclohexanone. 

This difference in catalytic activity may also be associated with the dispersion of rhodium on SBA-15 and SBA-16; by the catalytic results obtained and the XDR spectra, the rhodium has a greater dispersion onto SBA-15; and also considering the specific area BET, pore volume and size diameter, the phenol reacts more in the structure of the catalyst Rh-SBA-15.

**Scheme 1:**
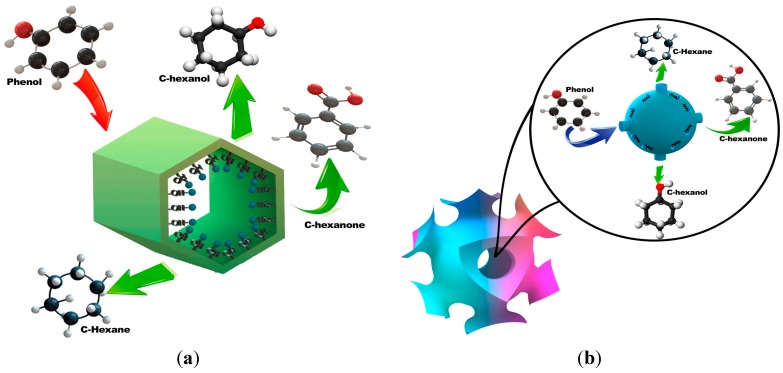
Reaction between phenol and SBA-15 and SBA-16. (**a**) SBA-15; (**b**) SBA-16.

In Scheme 1a, a schematic representation for the catalytic processes between phenol and Rh/SBA-15 catalyst is shown. The input and output of reactive and products are shown. In Scheme 1b, the entrance of phenol and its various catalytic products is also represented for the catalyst Rh/SBA-16.

**Figure 8:**
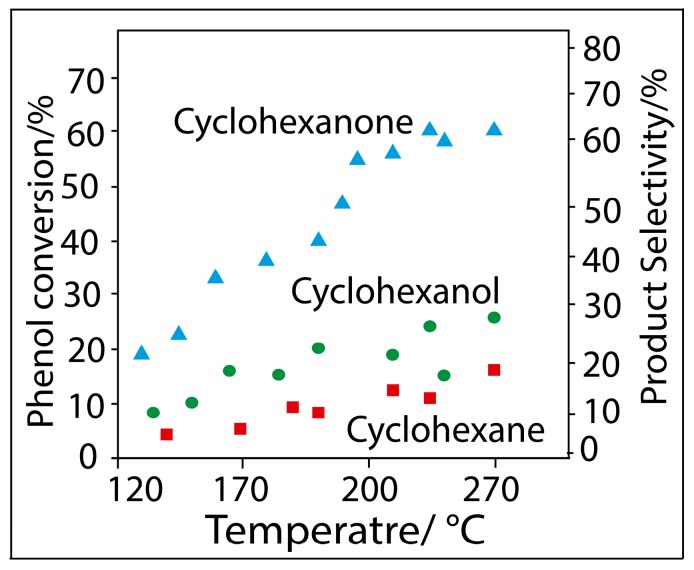
Effect of temperature on the hydrogenation of phenol with Rh/SBA-15.

In Figure 8, the effect of temperature on catalytic performance over Rh/SBA-15 (with 1 wt % in Rh) is shown. 

Reaction conditions: =180 °C. benzene/phenol = 2.0; H_2_/phenol = 6; weight catalyst = 0.6 g; WHSV = 27 mol·h^−1^·kg^-1^ (catalyst)^−1^.

A higher phenol conversion is obtained at 130 °C; the conversion decreased monotonously with the temperature. However it is noteworthy that the catalytic activity is higher with the catalyst Rh/SBA-15. It should be noted that Neri *et al*. [47] and Chen *et al*. [48] have also observed a maximum phenol conversion around 160 °C over Pd supported on alkali-doped Al_2_O_3_ and MgAl-CHT, respectively. With increasing temperature there is a decrease in the conversion of phenol caused by the lower presence of the reagent on the surface of the solid. Mahata *et al*. [10] on the other hand, observed a maximum phenol conversion around 230 °C over Pd/MgO catalyst; here the decrease in phenol conversion with increasing temperature has been attributed to thermodynamic limitations. In order to ensure that the reaction took place in the vapour phase, in this study the operative conditions already published in the scientific literature were adopted, *i.e.*, avoiding reaction temperatures below 120 °C since the boiling point of phenol is around 180 °C. The reaction temperature has a marked influence on the product selectivity as well. A nearly equimolar amount of cyclohexanone and cyclohexanol, together with traces of cyclohexane, are produced at 125 °C. The selectivity of cyclohexanone increases with an increase in the reaction temperature, while the selectivity of cyclohexanol decreases for both Rh/SBA15 and Rh/SBA16. Similar results were also observed over Pt- and Pd-supported Al_2_O_3_/MgO catalysts [10,17] and also over Ni/SiO_2_ catalysts [22]. However, in the case of Pd/MgAl-CHT catalysts the selectivity of cyclohexanone decreased with increasing temperature [49]. In the present study, the selectivity of cyclohexanone also increased to some extent up to 180 °C and then dropped off. The temperature 180 °C was thus considered optimum and subsequent studies were performed at this temperature. 

**Figure 9:**
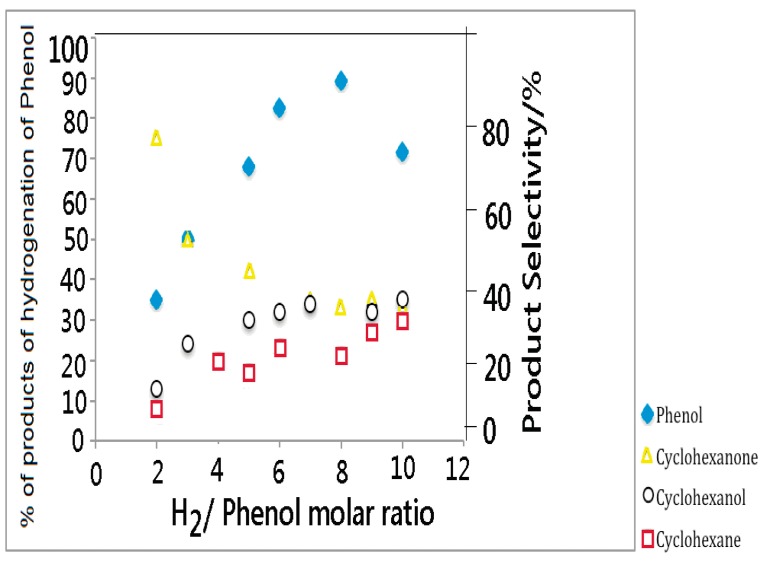
Effect of H_2_/phenol molar ratio on phenol conversion with Rh/SBA-15.

Figure 8 also shows that the reaction temperature has a profound influence on the product selectivity as well. A nearly equimolar amount of cyclohexanone and cyclohexanol, together with traces of cyclohexane, are produced at 130 °C. The selectivity of cyclohexanone increases, while that of cyclohexanol decreases with increasing reaction temperature. In the present study, the selectivity of cyclohexane also increases to some extent up to 180 °C and then levels off. The temperature of 180 °C was thus considered optimum and subsequent studies were performed at this temperature. The effect of H_2_/phenol molar ratio on catalytic performance of Rh/SBA-15, resulting as the better catalyst, is shown in Figure 9. 

Reaction conditions: =180 °C. benzene/phenol = 2.0; H_2_/phenol = 6; weight catalyst = 0.6 g; WHSV = 27 mol·h^−1^·kg^−1^ (catalyst)^−1^.

**Figure 10:**
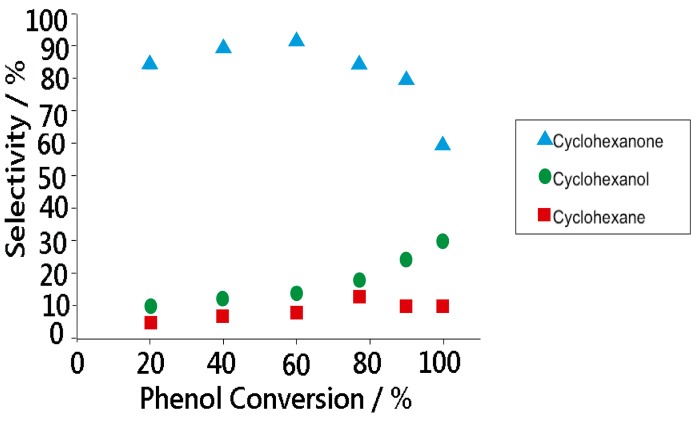
Phenol conversion in function of selectivity for hydrogenation over Rh/SBA15.

The phenol conversion is sensitive to H_2_ partial pressure. The conversion increases dramatically from about 35% for H_2_/phenol = 2 to above 90% for the H_2_/phenol = 9. However, a further increase in H_2_ partial pressure has a detrimental effect on the phenol conversion. The increase in phenol conversion is accompanied by a complete hydrogenation of phenol to cyclohexane. At the lowest H_2_/phenol ratio (*i.e.*, equal to 2), the selectivity of cyclohexanone and cyclohexanol are about 75 and 35%, respectively. The selectivity for cyclohexanone decreased monotonously, while the selectivity of cyclohexane decreased, when the H_2_/phenol ratio was increased. The selectivity of cyclohexanol, on the other hand, is insensitive to the H_2_/phenol ratio and it remains close to 30% in the entire range of H_2_/phenol ratios examined. In order to understand the reaction pathway by which the phenol hydrogenation proceeds over Rh/SBA-15, the phenol conversion versus selectivity was analysed, as shown in Figure 10. 

Reaction conditions: =180 °C. benzene/phenol = 2.0; H_2_/phenol = 6; weight catalyst = 0.6 g; WHSV = 27 mol·h^−1^·kg^−1^ (catalyst)^−1^.

It can be seen that cyclohexanone is the predominant product with a selectivity of more than 90% at low phenol conversions. As the conversion increases to about 65%, the selectivity of cyclohexanone declines at the expense of the selectivity for cyclohexanol. Above this conversion level, in addition to cyclohexanol, cyclohexane and traces of cyclohexene are also produced and their selectivity increases with the phenol conversion. This is consistent with results previously reported in the scientific literature [49]. The results show that the cyclohexanone is the main product of the phenol hydrogenation, and by a subsequent hydrogenation of this cyclohexanol is obtained. The cyclic hydrocarbons, cyclohexane and cyclohexene can be produced by dehydration and hydrodeoxygenation of the above.

**Figure 11:**
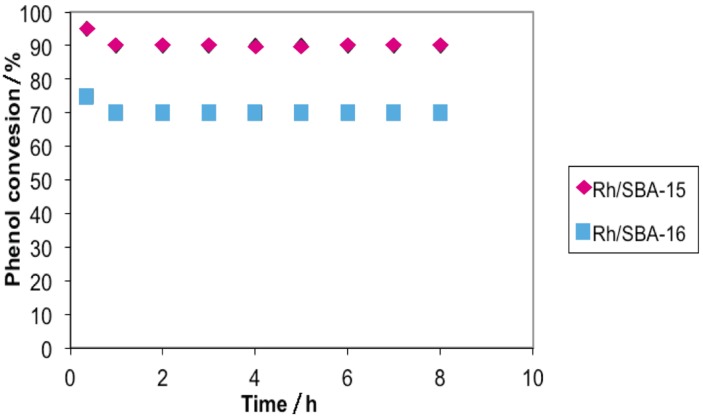
Effect of contact time on catalytic performance in the hydrogenation of phenol over Rh/SBA-15 and Rh/SBA-16, Temperature = 180 °C; benzene/phenol = 2.0; H_2_/phenol = 6; WHSV = 27 mol·h^−1^ kg·catalyst^−1^.

Figure 11 shows the phenol conversion at T = 180 °C as a function of time-on-stream over Rh/SBA-15 and Rh/SBA-16 catalysts. In both cases, there is only a minor decrease in the phenol conversion (within 10%) over the period of study (8 h). This minor deactivation does not alter the selectivity of cyclohexanone, which remains unchanged. If the selectivity of cyclohexanone can be improved to a satisfactory level by altering the composition of the support, this will probably be a good alternative catalyst for industrial production of cyclohexanone via phenol hydrogenation. 

The effect of the nature of solvent for phenol on catalytic performance has also been investigated over Rh/SBA-15 and Rh/SBA-16 (not reported here). The use of cyclohexane or ethanol as a solvent to phenol in the hydrogenation reaction is already known in the literature [2,3,4,5,6,7,8,9,10,11]. As we mentioned in the Experimental Section (Section 3.3), the motivation for this study is to select a suitable solvent for phenol hydrogenation because the use of cyclohexane produced a considerable amount of benzene as a byproduct. Four different solvents, namely benzene, toluene, cyclohexane and ethanol, were tested under similar experimental conditions. On comparing the data collected over benzene, toluene and cyclohexane, one can conclude that a relatively higher conversion can be achieved using cyclohexane as a solvent. However, the cyclohexanone selectivity is the same in all three solvents, and its selectivity is not influenced by the nature of the solvent. An appreciable amount of cyclohexane is obtained only in the case of benzene (note that a part of cyclohexane might be formed from benzene), while only traces of cyclohexene are formed when using toluene as a solvent. On the other hand, a three-fold drop in phenol conversion is observed using ethanol as a solvent with the present catalyst system (Rh/SBA-15). The drop in phenol conversion could be due to a competitive adsorption of phenol and/or H_2_ and ethanol molecules on the same active site. Phenol can also undergo alkylation reactions to produce the corresponding alkyl phenols over the catalyst, if alcohols are used as a solvent [30,31,32,33]. Hence, alcohols (methanol and ethanol) are not suitable solvents for phenol in the hydrogenation reaction over the Rh/SBA system.

## 3. Experimental Section

### 3.1. Sample Preparation

For the synthesis of mesoporous materials, a dedicated reactor was designed in our laboratory [39]. The reactor was constructed with Teflon and was provided with a controllable agitation system and pressure gauge. A safety valve was also installed, which enables the system to release excess pressure automatically, balancing the internal and external pressure [3,4,5,6,7].

#### 3.1.1. Synthesis of SBA-15

For the synthesis of SBA-15 [4,5,6,7,8], Pluronic P123 triblock copolymer (8 g, EO_20_PO_70_EO_20_) was dissolved in 2 M HCl (300 mL). Subsequently, tetraethyl orthosilicate (TEOS, 17 g) was added under vigorous stirring at 318 K. The molar ratio of the synthesis solution, TEOS:HCl:H_2_O:P123, was 1:5.87:194:0.017. After 7.5 h, stirring was stopped and the solution was aged for 15 h at 353 K. Then, the resulting white powder was filtered, washed, and dried. Finally, the powder was calcined by heating from ambient temperature to 823 K for 6 h, using a heating rate of 1 K·min^−1^ [48,49].

#### 3.1.2. Synthesis of SBA-16

SBA-16 was prepared as described in [5]: triblock copolymer Pluronic F127 was added to an aqueous solution of HCl, and stirred at a certain temperature overnight. Then, TEOS was added to this solution under vigorous stirring. After 10 min of stirring, the mixture was kept under static conditions at the aforementioned temperature for 3–72 h. The resulting solid products were collected by filtration, washed with ethanol, dried, and calcined at 550 °C in air for 5 h. Preparation of SBA-16 crystals relies on precise control of the reaction temperature and the ratio of reactants. Typical synthetic conditions for TEOS:F127:HCl:H_2_O were 1.00:0.0041:5.00:180 at 301 K, to obtain 1.00:0.0053:4.10:150 at 305 K, resulting in a rhomb-dodecahedron shape [49].

#### 3.1.3. Catalyst Synthesis

Catalysts were prepared by impregnating SBA-15 and SBA-16 with an aqueous solution of RhCl_3_·3H_2_O via incipient wetness technique. The impregnated powders were dried at 60 °C overnight. Calcination was carried out at 490 °C for 12 h at a heating rate of 10 °C·min^−1^. The rhodium content was kept constant at 1 wt %. 

### 3.2. Sample Characterisation

The crystalline phases of the synthesised mesoporous materials were identified by X-ray diffractometry (XRD). Spectra were scanned from 2θ = 1.0° to 20°, at a rate of 1°min^−1^. Fourier transform infrared spectra (FT-IR) were collected on the samples from 400 to 4000 cm^−1^; the determination of inter-species bonding was carried out from the identification of functional groups in the infrared absorption spectra. Adsorption-desorption nitrogen isotherms were measured at the temperature of liquid nitrogen, using an Autosorb 3B (Quantachrome, Boyton Beach, MI, USA). The surface area was determined by applying the Brunauer-Emmett-Teller (BET) equation and specific pore volume was calculated according to the Barrett–Joyner–Halenda (BJH) theory [36,37,38]. Transmission electron microscopy (TEM) measurements were made on a JEM-3010 electron microscope (JEOL, Tokyo, Japan) with an acceleration voltage of 300 kV.

### 3.3. Hydrogenation of Phenol

The vapour phase hydrogenation of phenol was tested in a fixed-bed tubular glass reactor (10 mm i.d. × 250 mm) at atmospheric using 0.6 g of the catalyst in the temperature range 130–270 °C, as reported in literature [49]. Before their catalytic behaviours were evaluated, the catalysts (around 0.15 g) were reduced in situ at 598 K with a heating rate of 1 °C·min^−1^ under flowing hydrogen (50 cm^3^·min^−1^) for 10 h, then cooled under hydrogen to the reaction temperature. Preliminary experiments reported in the literature, using cyclohexane as a solvent indicated the formation of a significant amount (15–20 mol %) of benzene [49]. Consequently, a series of solvents such as benzene, toluene, *n*-hexane and ethanol were examined. A considerable amount of benzene formation was also observed using toluene or *n*-hexane as solvents. Hence, in order to avoid the formation of benzene in the phenol hydrogenation, in the present study benzene itself was used as solvent. The hydrogenation of benzene to cyclohexane and other compounds using several catalyst systems is well known in the literature [47,48,49]. A blank experiment without phenol under the same experimental conditions produced an amount of cyclohexane (10–15 mol %). However, the use of benzene as solvent for phenol hydrogenation is preferred because under the adopted experimental conditions, the hydrogenation of phenol is predominant if compared to the hydrogenation of benzene, as reported in the literature [43,44,45,46,47,48,49]. A premixed phenol and benzene with a benzene/phenol molar ratio = 2 was fed into the pre-heater by means of a micro-feeder (liquid flow rate = 3*.*0 cm^3^·h^−1^). H_2_ was used as carrier gas as well as reactant; its flow rate was adjusted using a mass flow controller in such a way that the H_2_/phenol = 6. Chromatographic analyses were performed with a HP PAS/5 (25 m × 0.32 mm × 0.52 µm) capillary column (Hewlett Packard, Palo Alto, CA, USA), on a Shimadzu GC-17 (Kyoto, Japan) gas chromatographer with flame ionisation detector. Data between one and two hours were registered, then a comparative analysis and an interpretation of the results was completed.

The phenol conversion (%) (% _phenol_) is given by:

Phenol conversion (%) = [Phenol]_in_ − [Phenol]_out_/[Phenol]_out_ × 100

while reaction selectivity (as a percentage) in terms of the others compounds (*i.e.*, cyclohexanone) formation can be represented by:

Selectivity (%) of Cyclohexanone = [Cyclohexanone]_out_/[Phenol]_in_ − [Phenol]_out_ × 100

## 4. Conclusions

Our study of hydrogenation of phenol over Rh supported on mesoporous SBA-15 and SBA-16 in showed that there an is appreciable catalytic performance in terms of phenol conversion, and cyclohexanone selectivity can be obtained over catalysts with 1 wt % Rh loading. Additionally, under identical experimental conditions, the catalytic activity over mesoporous SBA-15 as support is better than over SBA-16 as support. The results of this study show that the Rh/SBA-15 exhibits stable activity during on-stream operation, but produces a mixture of cyclohexanone (about 60%), cyclohexanol (about 35%) and cyclohexane (about 15%), with a phenol conversion of about 80% at 180 °C. Finally, Rh/SBA-16 produces principally cyclohexanone for both synthesized catalysts in this work. The difference determined selectivity in this work is probably related with the structure of synthesized catalysts and mechanism of adsorption of the phenol.
